# Impact of Social Determinants of Health on Utilization of TCM and CCM Services: Differences by Disability Status

**DOI:** 10.1093/geroni/igaf122.4357

**Published:** 2025-12-31

**Authors:** Eunji G Kim, Ellen McCarthy, Dae Hyun Kim, Lan Luo, Chan Mi Park

**Affiliations:** Marcus Institute for Aging Research, Boston, Massachusetts, United States; Hebrew SeniorLife, Boston, Massachusetts, United States; Hebrew SeniorLife, Boston, Massachusetts, United States; Marcus Institute for Aging Research, Boston, Massachusetts, United States; Hebrew SeniorLife, Harvard Medical school, Boston, Massachusetts, United States

## Abstract

Transitional Care Management (TCM) and Chronic Care Management (CCM) services aim to improve post-hospital and chronic condition care, but utilization varies across populations. Social determinants of health (SDOH) can contribute to disparities in access to these services, especially among beneficiaries with disabilities. We examined how SDOH factors relate to TCM and CCM utilization, stratified by beneficiaries’ disability status. Using 2016-2020 Medicare Current Beneficiary Survey data linked to fee-for-service claims, we conducted matched case-control studies (1:2 ratio) of TCM-eligible (n = 4,762) and CCM-eligible (n = 25,380) beneficiaries. Eight SDOH domains, derived from 26 survey items, were standardized and analyzed via conditional logistic regression, adjusting for demographics and comorbidities. For TCM, lack of healthcare access (adjusted OR [95% CI]: 0.85 [0.76-0.95]) and poor neighborhood environment (0.83 [0.75-0.93]) were associated with lower TCM utilization. The associations were stronger among those without disability than those with disability for healthcare access (0.80 [0.64-0.99] vs. 0.86 [0.74-0.99]), but stronger among those with disability than those without for neighborhood environment (0.81 [0.70-0.93] vs. 0.89 [0.74-1.07]). For CCM, economic instability (1.16 [1.01-1.34]) and lack of transportation (1.21 [1.05-1.40]) were associated with greater utilization of CCM. However, stratified by disability status, these associations were attenuated and not statistical significant. SDOH impacts TCM and CCM utilization differently, with distinct patterns by disability. TCM under-serves those facing environmental and access barriers, especially individuals with disabilities, while CCM reaches people who are socioeconomically disadvantaged and less affected by disability. Integrating SDOH screening into care planning and aligning TCM and CCM delivery may improve equitable care.

